# Employer Policies and Practices to Manage and Prevent Disability: Conclusion to the Special Issue

**DOI:** 10.1007/s10926-016-9655-0

**Published:** 2016-07-30

**Authors:** Chris J. Main, William S. Shaw, the Hopkinton Conference Working Group on Workplace Disability Prevention, Benjamin C. Amick, Johannes R. Anema, Elyssa Besen, Peter Blanck, Cécile R. L. Boot, Ute Bültmann, Chetwyn C. H. Chan, George L. Delclos, Kerstin Ekberg, Mark G. Ehrhart, Jean-Baptiste Fassier, Michael Feuerstein, David Gimeno, Vicki L. Kristman, Steven J. Linton, Chris J. Main, Fehmidah Munir, Michael K. Nicholas, Glenn Pransky, William S. Shaw, Michael J. Sullivan, Lois E. Tetrick, Torill H. Tveito, Eira Viikari-Juntura, Kelly Williams-Whitt, Amanda E. Young

**Affiliations:** 1 Research Institute for Primary Care and Health Sciences, Room 1.56, Keele University, Keele, N. Staffordshire, ST5 5BG UK; 2 Liberty Mutual Research Institute for Safety, Hopkinton, MA USA; 3 University of Massachusetts Medical School, Worcester, MA USA

**Keywords:** Employer, Disability, Disability management, Disability prevention, Research priorities

## Abstract

*Purpose* Research of employer policies and practices to manage and prevent disability spans many disciplines and perspectives, and there are many challenges related to stakeholder collaboration, data access, and interventions. The purpose of this article is to synthesize the findings from a conference and year-long collaboration among a group of invited researchers intended to spur new research innovations in this field. *Methods* A multidisciplinary team of 26 international researchers with published research in employer-based disability management or related fields were invited to attend a 3-day conference in Hopkinton, Massachusetts, USA. The conference goals were to review the status of current research of workplace disability management and prevention, examine its relevance for employer decision-making, compare conceptual frameworks or theoretical perspectives, and recommend future research directions. In this paper, we summarize key points from the 6 resulting papers, compare them with an earlier 2005 conference on improving return-to-work research, and conclude with recommendations for further overarching research directions. *Results/Conclusion* In comparison with the 2005 conference, a greater emphasis was placed on organizational and social factors, employer roles and responsibilities, methods of implementation, non-clinical approaches, and facilitating stay-at-work as well as return-to-work. A special panel of employer consultants and representatives who were featured at the 2015 conference reinforced the importance of organizational culture, leadership style, and financial decision-making strategies at the employer level. Based on the conference proceedings, we recommend that future research in this area should strive for: (a) broader inclusion of workers and workplaces; (b) attention to multilevel influences in the workplace; (c) a focus on social as well as physical aspects of work; (d) earlier employer collaboration efforts; (e) more attention to implementation factors; and (f) a broader assessment of possible outcome domains.

## Introduction

In the introductory article to this Special Issue, Shaw et al. [1] described the objectives of the October 2015 Hopkinton “think tank” conference meeting, and how this special issue was conceived and operationalized. Historically, much of the early research in work disability was viewed through the lens of clinical management, and many workplace barriers were originally conceptualized as principally biomedical or ergonomic in nature, involving the match or mismatch between easily measurable physical limitations and job demands. More recently, research has concluded the need for a biopsychosocial perspective on disability, not just in terms of clinical management, but also in terms of workplace communication and support. The explicit task of conference attendees was “to evaluate the state of the science and to set a future research agenda that might reignite collaborative studies and develop and evaluate novel workplace intervention strategies to prevent work disability”. The purpose of this concluding article is to offer a synthesis of the principal findings and recommendations from the six papers, contrast these with some of the recommendations made in a related special issue of the *Journal of Occupational Rehabilitation* in 2005, and offer some suggestions as to development of relevant research and implementation strategies.

## Key Points from the Papers in this Special Issue

In the first article, Kristman et al. [2] recommend multi-level assessment frameworks with consideration of four organizational levels: worker, workforce, supervisor and manager, each characterized by examples of workplace factors assessed at the particular level, by the implied nature of the disability and by the most appropriate type of intervention. They offer this as a useful blueprint linking conceptualization, measurement and choice of intervention and which from a research design perspective may suggest the need for multilevel analysis. They identify four models describing employer decision-making: biomedical, financial management, personnel management and organizational development, which illustrate not only differences in focus, but differences in objectives for intervention. This is illustrated clearly in the difference between the focus of the grey literature and the scientific literature. They suggest the construction of a conceptual framework built on three core variable domains: (a) barriers to work re-entry; (b) aversive nature of the work environment given health limitations; and (c) the appetitive value of the work environment to provide rewards and support. The authors conclude by recommending the incorporation of more advanced and multi-level approaches to analysis; the inclusion of small and medium enterprises; and the need to incorporate workplace factors from all of the relevant domains.

In the second article, Williams-Whitt et al. [3] compare and contrast the types of interventions described in randomized scientific trials and the strategies more commonly considered by employers with a view to identifying intervention gaps and research opportunities. The research studies on interventions targeting the organization or group level were able to address a broader range of potential workplace issues than were addressed through individual-level interventions, but research assessing psychosocial job demands and employer attitudes is noticeably lacking. In contrast, most of the grey literature focused on productivity: reducing disability costs and increasing profits. The authors also conclude that return-to-work (RTW) and stay-at-work (SAW) interventions are primarily driven by the dominant medical work disability paradigm rather than a psychosocial paradigm. Workplace intervention components in the scientific (Cochrane and non-Cochrane reviews) and grey literature mainly concern changes to workplace design, job design, and work organization but the authors suggest a need for a greater emphasis on other components such as the role of the supervisor in facilitating job change and RTW. The authors encapsulate the difference between worker-centered and workplace-centered perspectives in their distinction between a “culture of science” and a “culture of practice”. They suggest that the design of interventions should be more participatory and target, or at least take into account, the worker’s social environment.

Young et al. [4] examine typical workplace outcome measures assessed in DM research, and they recommend multi-level sampling in order to simultaneously address the needs of multiple stakeholders. They distinguish four types of outcome: (a) *working*, but experiencing health-related limitations; (b) *off work* due to health conditions; (c) *back at work*; and (d) *full withdrawal* from the labor force. Each of these are associated with specific metrics on the basis of six specific criteria. They identify measures which allow for the assessment of whether or not an intervention has been successful in terms of: helping a person stay at work; in decreasing the amount of work absence; or in returning workers to productivity. They recognize that at times the relevant metric may not be the final outcome, but the evaluation of change across time, captured as types of trajectories, or in terms of movement along stages of re-integration though a RTW process. The authors stress that since organizational policies and procedures, as well the psychosocial work environment, may play a role in the extent to which outcomes occur, outcomes need to take into account the *context* in which they occur. They recommend further research into measurement development, particularly of employee-employer interaction and worker-coworker interactions (for which measures are largely absent) and they conclude by advocating the integration of scientific and business perspectives, with agreement upon a basic set of outcome measures that could facilitate the development of a data base from which new programs could be compared via benchmarking.

Main et al. [5] explore the theories of implementation science and their potential for understanding employer uptake as part of future research protocols. They recommend adoption of the Consolidated Framework for Implementation Research (CFIR) [6], and they conduct a classification exercise assigning implementation issues from existing work disability prevention research to one of the CIFR’s four phases and context. The authors identify the need for a common terminology; the importance of careful measurement and evaluation; and the need to address implementation fidelity and quality improvement. They identify not only different types of intervention, but a wide range of context-specific influences which they classify under five major headings. They then give specific consideration to the importance of the employer’s perspective as captured in the grey literature. They recommend further consideration be given to the fields of occupational rehabilitation, organizational psychology and organizational development (including well-being) as providing possible answers as to *how* interventions might be more successfully implemented. The authors then present an ongoing case-study in which an attempt was made to design, develop, and implement an employer-sponsored intervention to facilitate return-to-work (RTW) after work injuries. The authors conclude: “A clear message from this review is that successful implementations need to be planned, with clear specification of the desired outcomes and careful measurement of both the outcomes and the factors which influence them”.

Pransky et al. [7] consider work disability from a life-course perspective; broaden the focus from its traditional musculoskeletal focus to chronic illness in general, and examine specifically the occupational impact of cancer (on which there is a paucity of research) and mental illness, as examples of chronic or recurring conditions that might challenge conventional workplace return-to-work practices. However, the nature of workplace involvement is often difficult to determine and they identify significant methodological weaknesses in many of the studies. They recommend that future research of work disability should focus on earlier identification of at-risk workers with chronic conditions, on the use of more innovative and permanent accommodation strategies matched to specific functional losses, on stronger integration of the workplace into on-going medical rehabilitation efforts, and on attaining a better understanding of stigma and other social factors at work. They identify the need for theoretical models that can guide workplace interventions for chronic conditions which vary in visibility, periodicity and impact on work, and perhaps including condition-specific education of supervisors and employers.

Ekberg et al. [8] draw attention to the changing nature of the workplace and the increased diversity of working practices and conditions, with the consequences for workers of growing job insecurity and work intensification. They identify particularly vulnerable groups, noting that we have relatively little knowledge about “special” workplaces and work conditions and their opportunities and incentives for work disability prevention. The authors address four facets of this new working environment: temporary working arrangements; the special problems pertaining to small and medium enterprises (SMEs); virtual/distance working, and lone working. They recommend that future interventions involve structural and organizational aspects, as well as workplace conditions and employment security, and state that changes in governmental policy and incentives to provide RTW support across the entire spectrum of work arrangements and employers, and over the life course of workers may be required. They conclude with a set of specific recommended research priorities.

## Contributions of the Special Panel

The recommendations of the conference attendees were supplemented by reflections and feedback from a special 5-person panel who had real-world experience working with or consulting to employers on issues surrounding work disability prevention. The panelists participated in an initial closed-door session to address the six principal questions of the conference, and then the panel held a subsequent 3-h discussion with the full research group. Their primary points were framed by their slide presentation shown in Table 1. All five panelists acknowledged the gap in translation science and strongly supported the need to translate research into practice. They observed that, in their experience, the beliefs and values of leadership often overpowered evidence-based practice; and that organizational decision-making was often influenced by a crisis or in response to market factors or legal requirements.

**Table 1:** Initial questions and corresponding discussion points presented by the Special Panel

Question posed	Initial bullet points presented by Special Panel
Q1: What aspects of the workplace influence disability?	Top leadership values and belief systems Supervisor beliefs/behaviors/education Alignment expectations Effectiveness of performance management De-medicalization of disability decision-making Employer resources and appropriate utilization (Employee Assistance Programs, Occupational Medicine clinic, Workability Coordinator, etc.)
Q2: What aspects of the workplace influence disability?	Employer ability/willingness to accommodate Job satisfaction/employee engagement Psychologically safe workplace Physical safety and job demands Integration of work-related vs non-work related disabilities Workability coordinator Policies/practices/job aids Access to and integration of data
Q3: What employer measures can be taken to manage, prevent, or accommodate disability?	Functional job descriptions Training Implementation/operational/control plan Network of trusted/informed providers who understand your workplace/job demands and needs Employee engagement strategies
Q4: How do employers know if their disability management efforts are effective?	Lost time days Restricted days Absenteeism (planned vs unplanned) Direct costs Indirect costs Replacement Lost productivity Return on Investment/cost effectiveness/cost utility/Cost benefit Performance of vendors and internal processes Timeliness Quality of interaction/experience (satisfaction) Written communications Variation at the individual, workgroup, facility level Sustainability of RTW (recidivism) Presenteeism Engagement, retention, recruitment
Q5: How are new disability management practices taken up and implemented among employers?	Align with health beliefs/values of CEO Program dependent on support of CEO Align with company culture Communication Building relationships/collaboration Assess organizational readiness Policies/procedures/job aids (tools) Train/Train/Train Performance accountability Transparent display of results and variation Budgetary incentives for supervisors to accommodate
Q6: What disability problems are most challenging for employers and how will these change with future population trends in the workforce?	Distinguishing medical problems from functional capabilities Aging workforce Behavioral health and substance abuse issues Chronic disease in the workforce Upfront investment with uncertain outcomes (Net Present Value) Valuing indirect benefits (engagement, retention, recruitment) Diversity of employment types (employee, contractors, volunteers) Flexible work schedules and telecommuting/home work Unpredicted absence and presenteeism Sandwich generation issues (paternity, maternity, childcare, eldercare

In general, employers tended to concentrate their efforts on factors under their immediate control and within their domain of experience, with the consequence that less attention was paid to psychosocial and cultural issues which they perceived as less controllable, and for which organizational benefits were more difficult to quantify. The panel stressed the importance of a shift in focus from the medical aspects of disease or illness to the functional abilities of the employee, with employers and supervisors as natural collaborators in the return-to-work (RTW) process (although the panel considered that outreach visits by physicians might be helpful). The panel recommended four specific research priorities: (1) the value of personal stories to persuade management networks; (2) the importance of beliefs and values of organizational leaders; (3) a focus on organizational readiness for change; and (4) the need to study personal efficacy rather than just cost-effectiveness of workplace strategies.

## Comparison with the 2005 Special Issue

To provide a comparison with earlier work, recommendations from the conference can be compared with the findings from a related conference (to improve return-to-work research) and resulting special issue that was published in the *Journal of Occupational Rehabilitation* in 2005 [9]. In 2005, the major focus of research was on musculoskeletal disorders, and this has been expanded as suggested [10] to include other chronic illnesses (cancer and mental illness and consideration of the life-course. In 2005, Pransky et al. [9] identified six themes as priority areas: early risk prediction; psychosocial, behavioral and cognitive interventions; physical treatments; the challenge of implementing evidence in the workplace context; effective methods to engage multiple stakeholders; and identification of outcomes that are relevant to both RTW stakeholders and different phases of the RTW process. With the exception of “physical treatments” the themes have all been major features of the 2016 special issue. There has been a particular focus as suggested also on new concepts and study designs, better measures of determinants and outcomes, and on advances in translational research. The need for greater stakeholder involvement and commitment, and methods to address the unique challenges of each situation are still required, but the inclusion of grey literature and the inclusion of the panel of industry consultants has gone some way towards this.

Linton et al. [11], following their review of prognosis research and risk identification, highlighted a number of key methodological and research issues which remain relevant in 2015. They highlighted the absence of a clear conceptual framework and definitional issues as hampering the design of studies and the interpretation of research questions. The 2016 issue has built on their framework of outcome evaluation to include process evaluation, a specific focus on research into implementation and knowledge translation, embedded within a broad conceptual framework (the CFIR model) and with a stronger focus on (sustained) re-integration into work in which health-related work compromise is understood as a consequence both of incapacitating symptoms and of organizational factors. However, other than consideration of economic metrics and the importance of return-on-investment, it has not been possible to give direct consideration of the influence of the job market, although the importance of diversity and differing conditions of work specifically has been recognized in one of the contributions to this Special Issue [8].

Sullivan et al. [12] offered a review of scientific literature on psychosocial and behavioral interventions and work disability. They noted that such interventions had focused on psychosocial risk factors that exist primarily within the individual (e.g., pain catastrophizing, beliefs, expectancies) but that successful disability prevention required methods to assess and target psychosocial risk factors “outside” of the individual (e.g., interpersonal conflict in the workplace, job stress, etc.) using cost-effective, multipronged approaches. This recommendation for a contextual view has been one of the defining features of the 2016 issue but little research to explore interactions among different domains of psychosocial risk factors in relation to RTW outcomes has been undertaken in the last decade. Similarly, there has not been significant progress in challenges to effective secondary prevention of work disability, but the authors conclude that “effective secondary prevention of work disability will require research to develop cost-effective, multipronged approaches that concurrently target both worker-related and workplace psychosocial risk factors.” This remains as important an issue in 2016 as it was in 2005.

Loisel et al. [13] focused specifically on the importance of implementing evidence and acknowledged its complexity, and that implementation of evidence in work disability was a major challenge because many barriers existed, and many stakeholders were often involved.

They noted that intervention recommendations are often imprecise and not yet practical for immediate use. They found evidence for both clinical and non-clinical interventions in reducing work absenteeism. They stressed the need to involve all relevant stakeholders and to develop strategies which were effective and efficient, with the potential for successful implementation; based upon a clearer conceptualization of the broader context and on inter-relationships that determine return to work outcomes. There has been an attempt to do the former in the Australian case study presented in Main et al. [5], and the latter in the CFIR implementation framework described in the same paper.

Franche et al. [14] noted poor documentation and the use of diverse paradigms when implementing and studying workplace-based RTW interventions. Following their analysis of RTW stakeholder interests they considered that although friction was inevitable; it was possible to encourage stakeholders to “tolerate paradigm dissonance” while engaging in collaborative problem solving to meet common goals. Recommendations for future research included developing and recommended methods for engaging stakeholders and determining the optimal level and timing of stakeholder involvement. This topic was not a specific focus for discussion in the 2016 papers, although if some sort of framework for implementation is adopted (as recommended in Main et al. [5]), it may be possible to explore this further. The final recommendation of expanding research to work in diverse setting is discussed specifically in one of the papers in this Special Issue [8].

Franche et al. [15] found from their review of workplace based RTW interventions *strong evidence* that work disability duration was significantly reduced by work accommodation offers and contact between healthcare provider and workplace; and *moderate evidence* that it is reduced by interventions which include early contact with worker by workplace, ergonomic work site visits, and presence of a RTW coordinator. There was some evidence also for reduction in costs associated with work disability duration, but insufficient or limited evidence for sustainability of the effects for sustainability and evidence regarding the impact of the intervention components on quality-of-life. They concluded that although there was evidence that workplace-based RTW interventions could reduce work disability duration and associated costs, evidence regarding their impact on quality-of-life outcomes was much weaker. In this Special Issue, Williams-Whitt et al. [3] conclude that enhanced success in the outcome of work disability management will require a change in the type and focus of interventions, with more specific targeting of the social context of work.

Young et al. [16] recognized that reaching consensus on outcome among stakeholders had to be viewed in the light of other, sometimes competing, goals and the environments in which stakeholders operate clear definitions and criteria for evaluation were of key importance. The measurement of outcome was further considered in the current Special Issue [4] in their recommendation of four types of outcome: each appraised typically by specific criteria and inclusion of evaluation of change across time, through the RTW process. They recommended further work into measurement development, particularly of employee-employer interaction and worker/co-worker interactions (for which measures are largely absent). The measure of social interaction and appraisal of its significance in disability management remains a challenge which has not as yet been fully addressed, but the final 2005 recommendation of the integration of the scientific and business perspectives has been a core theme in the 2016 special issue.

In a companion paper, Young et al. [17] presented RTW as an evolving process, comprising four key phases: off *work, work re*-*entry, work retention*, and *work advancement*. They considered that the adoption of multiple phase-specific outcomes including a focus on incremental milestones might facilitates intervention choice and evaluation.

Finally, Loisel et al. [18] reported an observational study on collaboration between members of an interdisciplinary team discussing workers absent from work due to musculoskeletal disorders. They found that various factors influence collaboration between the rehabilitation team and the stakeholders but that in general, stakeholder endorsement of the team’s therapeutic principles and confidence in their approach emerged as particularly important factors, and that diverse strategies, most often, education and awareness-raising, were used by the team to foster collaboration among the parties. Since that time, there have been a number of qualitative studies on the health/work interface [19–21] and there may be a place for further such targeted studies to identify influences on implementation.

## Conclusions and Recommendations

There have been important changes in the world of work which have implications for the management of work disability. There is increasing diversity in the types of work, with differing conditions of employment and working circumstance; in the workforce, with sociodemographic changes and economic drivers that require people to work to a greater age and thereby carrying an increasing burden of age-related symptoms for which accommodation is required. Perhaps the most important change however is that the objective of remediating all forms of disability (whether through medical treatment or by finding biomechanically-based ergonomic solutions) before return to the workplace no longer appears realistic and that new solutions are required to facilitate RTW and sustain work engagement, despite ongoing symptoms. Therefore, future research should include a broader spectrum of workers, including those who are struggling to stay at work but have not filed for disability benefits.

Moving from a science of work disability to a science of disability management permits a re-alignment in primary outcome from restoration of function to the more challenging outcome of sustained re-engagement in work. It has been suggested that the challenge of work disability should be re-conceptualized as a problem of sustained re-integration into work; a term which can still incorporate traditional perspectives on work disability, but supplement those with a contextual analyses of the nature of the workplace as a potential determinant of successful re-integration into work. However this focus necessitates the involvement of all interested parties rather than exclusive outsourcing to healthcare personnel. This reconceptualization of work disability suggests the need for a re-energized and refocused research agenda as a means of clarifying the determinants of response to interventions, tackling the determinants of behavior change in interventions, improving the management of work disability, engaging all relevant stakeholders, and improving outcomes as gauged by relevant and agreed metrics. In particular, future research should attend to the social and multilevel influences of policy makers, managers, supervisors, co-workers, insurers, and case managers, all of whom impact on RTW and SAW strategies and outcomes.

There are a number of implications which derive from this reconceptualization. First, such a conceptualization invites consideration of the determinants of behavior change at each of the levels and places the process of implementation at the center of the stage. Second, the influences on implementation then become part of the design of the intervention, rather than processes simply as confounders of treatment outcome. We hope that this shift in focus will encourage the design and development of a strategic approach to work disability, facilitated by deconstruction of the challenge of work disability into the series of linked problems which will need to be addressed if the ultimate objective of sustained RTW is to be achieved. Third, this perspective facilitates the inclusion of both worker-centered and workplace-centered initiatives into the re-integration process, perhaps stimulating a fundamental change in organization culture, including a focus on work retention, as a means of preventing unnecessary disability. Fourth, if work disability is recast as a problem of sustained re-integration into work, a broader concept of relevant science and research methodology, informed by additional insights from the fields of organizational psychology and organizational development, becomes relevant.

With this broadening of the concept of work disability, the stages of implementation thus become mini-interventions in their own right, facilitating shared output by interested parties, and crossing the interface between science and business. Such collaborative research efforts may offer the opportunity for specific research studies (perhaps including qualitative approaches), recognizing that the nature of the research environment seldom offers the conduct of an RCT except at a macro level. Therefore, future research should include assessment of organizational variables that are likely to be critical factors in whether or not a RTW or SAW strategy is adopted.

Given the marked diversity in workplaces, workforces and health-associated work compromise, consideration of the particular context is of paramount importance. We can only offer therefore a general picture of how the design of an intervention might be approached (specifics are contained in the individual papers). Prior to introducing interventions we advocate a *re*-*engagement analysis* beginning with consideration of possible interventions [3]; taking into account the characteristics of the targeted population [7]; factoring in differences in the type of work and working conditions [8]; proceeding with identification of all stakeholders, blending the recommendations of Franche et al. [14] and Young et al. [16], applying recent advances in implementation science as to how the intervention might be implemented [5], and taking into account the need for careful and appropriate measurement at every stage of the process [4]. Therefore, future research should involve earlier stakeholder collaboration efforts with employers to design interventions that can be more feasibly woven within existing operations and to include a broader assessment of outcome domains that will be useful to demonstrate a positive return-on-investment.

The involvement of the industry panel in the 2015 conference in our view represents an advance on 2005 and in so doing we have perhaps narrowed the “distance” between the worlds of medical science and business. In attempting to incorporate at least a sample of the grey literature, in conjunction with the scientific literature, we believe we have obtained a broader view of the complexity of work disability and disability management. Although there have been examples scattered around the literature of case studies in which employers have been directly involved and there have been studies for the last 15 years specifically addressing aspects of employer engagement (such as the role of line managers in work disability management), it is difficult to know how to integrate findings from such diverse sources and working circumstances, but a number of steps seem to suggest themselves. First, we might usefully return to Franche et al. [14] and Young et al. [16] to develop a set of research priorities into the role and function of stakeholders with a view to develop not only agreed outcomes for interventions but the nature of their engagement with the implementation process, thereby clarifying their role not only in RTW but is sustained re-engagement in the working environment. Second, we might identify the behavioral changes required by both the organization and the “injured” worker and construct a multifaceted intervention. Third, we could examine the interdependence of the behavioral changes among the various stakeholders and develop a phased implementation strategy for which valued outputs (some of which may be very simple) can be obtained at each stage and agreed by all parties.

Perhaps the strongest message from this special issue is that in devising successful interventions for work disability, implementation needs to be considered, with clear specification of the desired outcomes and careful measurement of both the outcomes and the factors that influence them. The key stakeholders must find ways of working collaboratively to choose relevant outcomes for workers, employers, and other stakeholders. Successful design and implementation of workplace disability management practices may require a broader theoretical framework than hitherto acknowledged. It now seems clear that effectiveness of occupational interventions for work disability is unlikely to improve without attention to the *context* where the intervention takes place and to facilitate implementation. Research into work disability hitherto has tended to disregard such factors as of marginal relevance at best, yet they may be relevant not only as obstacles to implementation but as factors which need to be addressed as part of the whole research strategy. Broadening the focus of effort represents a major research challenge, but encouragement has been found in increased understanding of the determinants of behaviour change at both the individual and organisational level.

For several decades research into the moderators and mediators of outcomes in clinical medicine has moved from a narrow biomedical model to a broader biopsychosocial model [22], advocated in the occupational field many years ago by Feuerstein [23] and later by Loisel et al. [24]. In consideration of work disability management, it is perhaps now time to fully embrace this perspective not only in conceptual terms but in terms of interventions, their implementation and our overall research strategy.
